# Mania Following Bereavement: State of the Art and Clinical Evidence

**DOI:** 10.3389/fpsyt.2020.00366

**Published:** 2020-05-06

**Authors:** Claudia Carmassi, Katherine M. Shear, Martina Corsi, Carlo Antonio Bertelloni, Valerio Dell’Oste, Liliana Dell’Osso

**Affiliations:** ^1^Department of Clinical and Experimental Medicine, University of Pisa, Pisa, Italy; ^2^Columbia University School of Social Work, New York, NY, United States

**Keywords:** funeral mania, bereavement, complicated grief, bipolar disorder, manic episode, manic onset

## Abstract

Bereavement is the state of loss, determined in most of the cases by the death of a close person. It is probably the greatest sorrow that can occur in an individual life. Grief is a normal, healthy response to loss, evolving through stages in the process of mourning. In some cases, bereavement may lead to the outburst of manic episode: despite literature data being scarce, reports have explored this important clinical entity, variously called as “funeral mania” or “bereavement mania”. We systematically reviewed the literature exploring the possible relationships between bereavement and the onset of a manic episode, both first or recurrent pre-existing episode, besides describing a case report on a manic episode in the aftermath of a loss event, with an accurate evaluation of prior mild mood spectrum instability, supporting the role of loss-events as potential risk factor for bipolar illness progression. This article tries summarizing existing evidence on the debate whether clinicians should consider mania as a possible bereavement reaction.

## Background

Increasing interest has been recently devoted to psychopathological reactions to loss and evidences have supported the existence of a specific clinical diagnosis addressing about 7–20% of subjects who develop long-lasting symptoms of intense grief, that interfere with adaptation and re-engagement in life, differently named as Complicated Grief (CG), Traumatic Grief or Prolonged Grief Disorder (1, 2), characterized by persistent desire and pervasive yearning of the deceased person, deep pain and frequent crying or worrying (3–8). The DSM-5 introduced, for the first time, the diagnosis of “Persistent Complex Bereavement Disorder (PCBD)” in the section for conditions and criteria needing further research (9). The death of a loved one may also trigger the onset or the worsening of mental disorders, particularly Major Depression Disorder (MDD) and Post-Traumatic Stress Disorder (PTSD), however different reactions have been described in the literature in the framework of a loss event, such as mania (10, 11). This is particularly interesting, also considering the possible partial clinical and neurobiological overlap between grief reaction, mood disorder and PTSD (12–14).

The outburst of a manic episode after the death of a close one has been called “funeral mania” or “bereavement mania”. The temporal relationship between the loss and the onset of a mental disorder may vary from this later occurring immediately after the loss or later on as an anniversary reaction. “Funeral mania” refers to a typical manic episode occurring within one week from the death of a close relative or friend, while “bereavement mania” is considered to be a kind of psychogenic mania that emerges in a short time following the death of a closed one (11, 15). Although only anecdotal cases have been reported on manic reactions after the death of close kinsmen data seem to reveal that a loss event can be either the trigger of a first manic episode in patients with a negative psychiatric history, or a predictor of manic relapse in patients with a history of bipolar disorder (BD) (16). Most of the literature, in fact, has been devoted to depression leading to important acknowledgments in most recent nosography related to the interplay between psychopathological reactions to relevant loss events and mood reactions. In this regard, one of the most important results was that the DSM-5 first adopted the “bereavement exclusion concept” in the mood depressive episode allowing to diagnose a major depressive episode independently of a specific time lapse in the aftermath of a relevant loss. Scant data, however, have focused attention on bipolar onset with a manic episode in the aftermath of a relevant loss, despite the observation of mania in response to traumatic events has been discussed in the literature (15); yet the speciﬁc mechanisms for this association remain unclear.

The purpose of the present manuscript is to summarize papers linking bereavement with bipolar onset with a manic episode or with a first manic episode in the context of a mood disorder, suggesting the need to deserve attention to this possible outcome. Secondly, we also reported a case description of a case followed in our clinic in which we examined a lifetime mood spectrum, with a mild mood instability and “mild manic states” as vulnerability, leading to consider reflecting on the role of loss-events as risk factor for illness progression.

## Methods

### Search Strategy

Medline, PubMed and Scopus databases were accessed in order to research and collect English language papers published between January 1st, 1960 and November 1st, 2019. Free text terms and MeSH headings for the topics of bereavement and manic episode or bipolar disorder were combined as it follows: ‘(“funeral mania” OR “bereavement” OR “complicated grief”) AND (“manic episode” OR “bipolar disorder”)’.

### Eligibility Criteria

We included in the present manuscript case reports published in English in indexed journals and when full manuscript was available. The following inclusion criteria were adopted: case reports or articles reporting manic episode onset in the aftermath of a significant loss in patients with no previous mood disorder or first manic episode in the context of a mood disorders after bereavement.

### Study Selection

**Figure 1:**
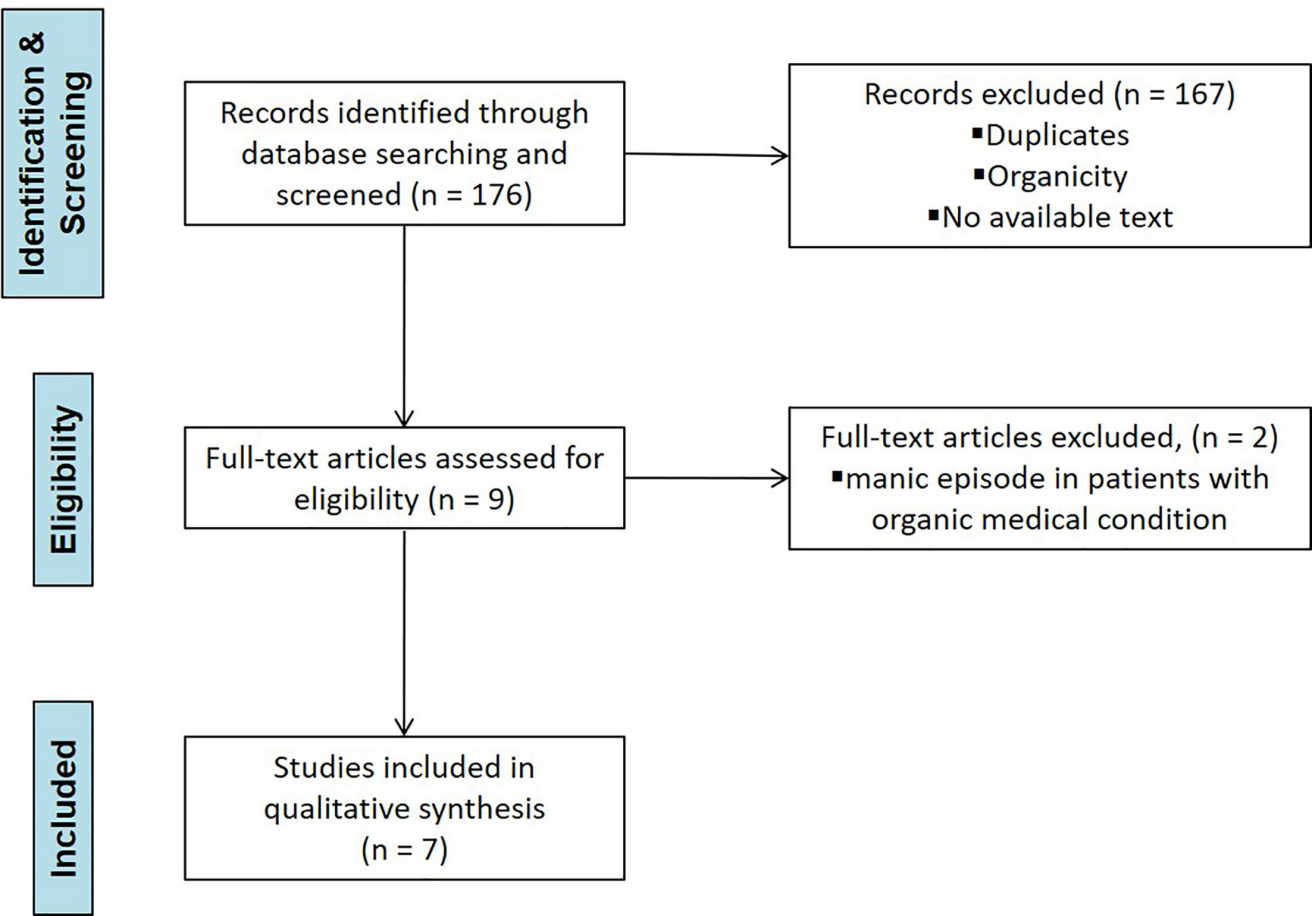
PRISMA flowchart of study selection process.

Each study was screened for eligibility by the Authors after reading the title and abstract. Any uncertainties concerning eligibility were discussed and resolved among all authors. The decisions for inclusion or exclusion are summarized in a flow chart according to Preferred Reporting Items for Systematic reviews and Meta-Analyses (PRISMA) recommendations. Details are reported in **Figure 1**.

## Results

### Process of Study Selection

The electronic database search retrieved 176 potential results and Authors equally contributed in identifying potential information specific to this topic amongst the titles and abstracts of the publications. The first selection excluded 167 titles because: a) duplicates; b) not concerning the scope of the paper; c) not informative enough/no full manuscript available. Nine articles were identified as potentially relevant after screening of titles and abstracts. These studies were assessed for eligibility in full text. The second selection excluded two articles after being read and reviewed, as the information reported manic episode onset in the aftermath of bereavement in patients with an organic medical condition. Finally, seven papers were included in the present manuscript, reporting 15 case reports: three of these reports were excluded after being completely read and evaluated, as they describing manic relapse following bereavement in patients with a diagnosis of Bipolar Disorder (16–22). Key characteristics of included studies are summarized in **Table 1**.

**Table 1:** Case report studies reporting first manic episodes in the aftermath of a significant loss in patients a) without any previous lifetime mood episodes or b) with previous depressive episodes only.

A) PATIENTS WITHOUT ANY LIFETIME MOOD EPISODE						
Study	Patient (gender)	Age (years)	Relationship with the deceased	Description		Treatment
				Index episode		
Rickarby (16)	Male	55	Son (killed)	Acute Manic episode followed by recurrent manic episodes yearly in the anniversary		Hospitalization: Neuroleptics and lithium
	Male	25	Mother	Acute Mania with psychotic symptoms		Hospitalization: lithium
Gill (17)	Female	49	Husband Mother (4 years later)	Acute mania Acute Mania with psychotic symptoms		Hospitalization: neuroleptics and ECT Hospitalization: neuroleptics
	Male	46	Mother Father (2 years later)	Acute Mania Acute Mania		Hospitalization: no treatment data Hospitalization: no treatment data
	Female	24	Father (suicide)	Acute Mania		Hospitalization: no treatment data
Morgan et al. (18)	Female	37	Husband	Acute Mania with psychotic symptoms		Hospitalization: Neuroleptics
Carmassi et al. (19)	Female	52	Son	Acute Mania with psychotic symptoms		Hospitalization: Neuroleptics and mood stabilizers
**B) PATIENTS WITH PREVIOUS DEPRESSIVE EPISODES ONLY**						
**Study**	**Patient (gender)**	**Age (years)**	**Relationship with the deceased**	**Description**		**Treatment**
				**Previous history**	**Index episode**	
Rickarby (16)	Female	44	Husband	Severe depressive episodes (responsive to imipramine)	Acute Mania	Hospitalization: lithium
	Female	21	Father	Severe depressive episodes with following recurrences and periods of mood instability (no mania)	Acute Mania	Hospitalization: lithium
Hollender and Goldin (20)	Female	44	Son	Recurrent depressive episodes (the first occurring after the loss of her husband 3 years earlier)	Acute Mania with psychotic symptoms	Hospitalization: TCA and mood stabilizers
Ranga et al. (21)	Female	34	Husband’s cousin	Recurrent major depressive episodes	Acute Mania with psychotic symptoms	Hospitalization: neuroleptics
Rosenman and Tayler (22)	Female	28	Husband	Post-partum depression	Acute Mania	Hospitalization: no treatment data

ECT, electroconvulsive therapy; TCA, tricyclic antidepressants.

### Characteristics of Included Studies

Out of the 12 cases we found in literature, nine cases were women, while only three were men. The mean age was 38.25, with the youngest being a 21 years old female and the oldest a 55 years old male. Four out of the 12 case presented their onset during the second and fourth decade of their life and two during their third and fifth decade. The most represented relationships among the cases were son and husband/wife, reported in three and four cases respectively. Four patients developed the symptoms after the death of their parents, two following the mother’s death and two after the deceased of the father. The remaining case concerned the death of another relative (husband’s cousin). The most frequently reported cause of the death was by accident (five out of 12 cases), followed by sudden death and cancer (two cases each). In three case the cause of death was not reported. A negative psychiatric clinical history was found only in seven out of the 12 cases. The remaining five cases all reported a positive history of Major depressive disorders without previous manic episode or Bipolar history. Symptoms onset varied from a few hours/days to several years after the death. In one case, Rickarby (16) describes symptoms recurrence once every year, while someone of us published a case of a woman whose onset was on her son’s birthday (19).

### Case Presentation

We discuss the case of a 77-year-old patient with a diagnosis of BD hospitalized at the inpatient unit of the Psychiatric Clinic of the University of Pisa for a depressive episode, who experienced three years before a manic episode after his wife’s death.

Mr. XY was a 77-year-old Italian male, widower, graduated at the high school at the age of 18 and living with the first of his two daughters. His longitudinal evaluation revealed no family loading for psychiatric illness, with no complications at birth, pregnancy and delivery. No developmental disorder or delays were referred.

The onset of the psychopathological symptomatology seems to date back to the age of 18 when, following a stressful life event (death of his father), he presented low mood levels and elevation of anxiety, resolved spontaneously in few months. In the following years the patient experienced cyclical seasonal depressive mood and energies swings, with concomitant fluctuations of the anxiety levels; however, in these years, the patient did not present interferences in the social and work functioning, with no need for psychiatric care. In January 2010 (70 years) after diagnosis of bladder carcinoma, successfully treated, the patient experienced a period characterized by slight lowering of mood level, clinophilia and increased level of anxiety. The patient decided to contact our Psychiatric Clinic, where a Major Depressive Episode was diagnosed and a psychopharmacological therapy based on Pregabalin (150 mg/day), Quetiapine (25 mg/day), Paroxetine (20 mg/die) and Delorazepam (30 mg/day) was prescribed. Afterwards, Mr XY maintained a good psycho-affective compensation until the summer of 2014 when, subsequently an oncological disease, he lost his wife. In the following weeks he progressively showed an elevation of mood, high energy level, reduction in hours of sleep, greater search for pleasant activities (attending numerous partners, making several trips around the world), carrying out risky economic activities (dangerous real estate and commercial investments), managing by himself the psychopharmacological therapy. This period lasted about 18 months and was followed by an episode of referred low mood of about 2 months, for which he contacted again our Psychiatric Clinic; a new psychopharmacological therapy was settled, based on Pregabalin (75 mg/day) and Paroxetine (15 mg/day) with good clinical reward until 2017. In the following months Mr. XY presented depressive fluctuations of mood, energy and anxiety levels with the appearance of gastrointestinal somatizations at the epigastrium and alterations of body weight without sickness-free interval. Two hospitalizations were made in a short time frame in 2017 due to the symptomatology described above: the patient was treated by several psychopharmacological associations, reaching psychoaffective well-being with Valproic acid (300 mg/die), Aripiprazole (2.5 mg/die) and Venlafaxine (75 mg/die).

## Discussion

To the best of our knowledge, only four papers have reported about the onset of one’s first lifetime manic episode in the aftermath of the loss of a significant other, as well as four papers described first manic episode following grief in patients with previous depressive episodes only. Several studies have reported a correlation between stressful life events and the onset or the recurrence of mood disorder. Severe negative life events, in fact, have been found to be associated with more than four times the risk of relapse and an increase up to three times in illness duration. These same events have also been found to predict the onset of mood disorders among children of people with a history of BD (23–25). In recent years, researchers have begun to consider the influence of psychosocial variables on depression and mania separately. Major losses appear to specifically predict the course of depression within BD while the effects of negative life events on mania are less clear. Nevertheless, some studies analysing severe negative life events found a possible relationship between negative events and manic episodes, despite conducted on very small samples (11, 26–28). In one prospective study of undergraduates with bipolar spectrum disorders was found that life stressful events lead to a hypomanic symptomatology among those students with depressive cognitive styles (29).

The clinical features of mania following bereavement emerged by our review do not differ from the other types of mania. Authors describe changes in mood (euphoric), sometimes mixed with depressive feelings. Carmassi et al. (19) reported how the patient alternated elevated mood to affective instability and dysphoria. Authors often describe sudden mood switches from euphoria to hostility and aggressiveness (18). All cases reviewed are characterized by patients with an increase in energy levels, with lack of sleep, pressure to speech (often rapid and with flight of ideas), tendency to undertake many tasks and, in general, agitated and restless. Excessive spending and drinking were also reported. Despite scant literature data, a possible finding was hypersexuality with sexual disinhibition and flirting behaviors. Several kinds of delusions were reported: persecutory (family conspiring against the patient) (18), of “grandeur” (being a millionaire) (16), having special powers to help people, thought broadcasting (18). These patients often developed delusions about the deceased: from asserting to be still in contact with him/her, to talking to him/her, to deny the reality of the death (19).

Our case presented a patient with a previous history of depressive but not (hypo)maniac episodes, with a first manic episode in the aftermath of a loss event. Patient previous mood instability, characterized by exclusively depressive episodes, was carefully assessed during the hospitalization after maniac episode by using a specific questionnaire investigating lifetime mood spectrum symptoms in a dimensional perspective, including atypical or not full-blown symptoms of mood, called MOODS-SR (Mood Spectrum-Self Report) (30, 31). Symptoms of (hypo)maniac or mixed mood instability resulted absent or subthreshold and did not reached clinical relevance until the loss event happened. As previously reported in some other reports (16, 19), these patients appear primarily to be suffering from affective instability that does not meet criteria for a classic manic, and often seem to be display symptoms consistent with a mixed episode in the aftermath of a relevant loss. However, current DSM-5 nosography abolished the previous mixed episodes classification, replacing it with a mixed-features specifier that can be applied to episodes of major depression and (hypo)mania (9). Therefore, it could be useful reflect on the role of loss-events as risk factor for illness progression, detecting lifetime mood spectrum liability, also defined as mild mood instability and “mild manic states”, as a vulnerability to develop a full-blown manic episode.

Nevertheless, these reports do not provide an estimate of how common this phenomenon is. In a broader analysis of 1,565 Danish first psychiatric hospital admissions, authors (25) found that death of a mother or sibling was more common before an admission for mania than it was in the general population, but only for death by suicide. Given the heritability of suicide and bipolar disorder, though, people with bipolar disorder would be genetically more vulnerable to family suicide rather than people without bipolar disorder. Moreover, a USA study on 27,534 subjects found an association between unexpected death and the onset of manic episodes in a general population sample across the life course, suggesting that unexpected death of a loved one may be a substantial risk factor for the onset of a manic episode, especially among older adults, and even among those with no prior history of mood, anxiety, or alcohol disorders (32).

Although several studies found no direct effect of negative life events as a predictor of increases in manic symptoms (33, 34), it has been reported subjects may be vulnerable to increases in manic symptoms after negative life events (35–37). Independent severe negative life events have been reported to be more common in subjects who have consequently developed manic episodes rather than among healthy controls (38–40), though one study did not confirm it (41). Loss-related events, employment problems and financial difficulties have been reported more frequently before manic episodes with respect to depressive ones (42–44).

The casual relationship between mania onset and bereavement has been discussed in the literature, but with few evidences. Psychodynamic models focused on the manic defence hypothesis (45). In these models, mania was hypothesized as a flight from painful feelings. These models predict that mania will occur after negative life events, as a defensive reaction. Newer cognitive behavioral formulations have proposed that people with bipolar disorder might avoid focusing on threatening information (46). A terror management study provided some evidence for defensive reactions among people who are vulnerable to hypomania (47). The loss of social support, particularly through bereavement, has been hypnotized to create a loss of control and can trigger mania or depression, and (hypo)manic symptoms might facilitate new social connections, whereas disinhibited and risky behavior exhibited during mania can cause the breakdown of vital relationships (48). Furthermore, grief reaction often includes sleep disruption as well as more general schedule disruption that have been hypothesized as potential mechanism through which life stressors lead to increases manic symptoms (49, 50). Sleep disruption, indeed, appears to trigger manic symptoms, as evidenced by naturalistic and experimental studies (51, 52).

Even if “Funeral mania” is a known clinical entity, it has no nosological status in DSM and ICD. The loss of a loved one is a universal experience that we are all potentially exposed to and the grief that ensues in the immediate is, however painful, a physiological reaction.

## Conclusions

Bereavement could lead to several pathological reactions, whose most common, experienced by up to 7% of bereaved people, is CG (53, 54). Several authors agree on the possibility that, in a minority of cases, a pathological response to bereavement develops into CG, correlated to a significant and persistent impairment of the individual’s social-working functioning and a high suicidal risk (55, 56). As emerges from the analysis of the clinical cases mentioned above, bereavement may also be considered as a risk factor for a recurrence of a manic episode in people suffering of BD or, rarely, for the onset of a first manic episode in people without history of mental disorders. Thus, our ﬁndings should alert clinicians to the possible onset of mania in the aftermath of a significant loss either in healthy individuals or in patients affected by major depression shifting the main focus of observation from the early detection of a possible depressive onset to the eventual occurrence of a first lifetime manic episode, in order to provide adequate and prompt treatment.

## Data Availability Statement

The datasets generated for this study are available on request to the corresponding author.

## Ethics Statement

Written informed consent was obtained from the individual(s) for the publication of any potentially identifiable images or data included in this article.

## Author Contributions

All authors gave substantial contribution to the study and approved the final version of the manuscript and the manuscript submission to Frontiers in Psychiatry.

## Conflict of Interest

The authors declare that the research was conducted in the absence of any commercial or financial relationships that could be construed as a potential conflict of interest.
